# The first case of monkeypox in Hong Kong presenting as infectious mononucleosis-like syndrome

**DOI:** 10.1080/22221751.2022.2146910

**Published:** 2022-12-12

**Authors:** Kelvin Hei-Yeung Chiu, Shuk-Ching Wong, Anthony Raymond Tam, Siddharth Sridhar, Cyril Chik-Yan Yip, Kwok-Hung Chan, Nicholas Foo-Siong Chew, Kenyon Ka-Yun Man, Wan-Mui Chan, Jonathan Daniel Ip, Allen Wing-Ho Chu, Janice Yee-Chi Lo, Ivan Fan-Ngai Hung, Kwok-Yung Yuen, Kelvin Kai-Wang To, Vincent Chi-Chung Cheng

**Affiliations:** a Department of Microbiology, Queen Mary Hospital, Hong Kong Special Administrative Region, People’s Republic of China; b Infection Control Team, Queen Mary Hospital, Hong Kong West Cluster, Hong Kong Special Administrative Region, People’s Republic of China; c Department of Medicine, School of Clinical Medicine, Li Ka Shing Faculty of Medicine, The University of Hong Kong, Pokfulam, Hong Kong Special Administrative Region, People’s Republic of China; d State Key Laboratory for Emerging Infectious Disease, Carol Yu Centre for Infection, Department of Microbiology, Li Ka Shing Faculty of Medicine, The University of Hong Kong, Hong Kong Special Administrative Region, People’s Republic of China; e Department of Microbiology, School of Clinical Medicine, Li Ka Shing Faculty of Medicine, The University of Hong Kong, Pokfulam, Hong Kong Special Administrative Region, People’s Republic of China; f Department of Radiology, Queen Mary Hospital, Hong Kong Special Administrative Region, People’s Republic of China; g Department of Health, Centre for Health Protection, Hong Kong Special Administrative Region, People’s Republic of China; h Department of Infectious Disease and Microbiology, The University of Hong Kong-Shenzhen Hospital, Shenzhen, People’s Republic of China; i Centre for Virology, Vaccinology and Therapeutics, Hong Kong Science and Technology Park, Hong Kong Special Administrative Region, People’s Republic of China

## Introduction

Monkeypox is a zoonotic infection caused by monkeypox virus (MPXV). It was first described in human in 1970, with subsequent circulation in endemic areas such as central and west Africa [1]. Since 2003, MPXV emerged outside Africa, and caused the first major outbreak in the USA linked to imported exotic animals [2]. On 7 May 2022, a case of monkeypox in a traveller returning from Nigeria to the United Kingdom was reported, leading to the subsequent circulation of MPXV in predominantly sexually-active young males [3]. This outbreak led to more than 65,000 cases in at least 90 non-endemic countries on multiple continents [4]. The current human monkeypox outbreak was declared a Public Health Emergency of International Concern by the World Health Organization on 23 July 2022 [5]. Here we reported the first case of imported human monkeypox in Hong Kong in September 2022, who presented with infectious mononucleosis-like syndrome, as the newly described manifestation in the literature.

## Case report

A 30-year-old Chinese male was admitted to Queen Mary Hospital with sore throat and dysphagia when he returned to Hong Kong on 5 September 2022. He travelled to the USA from 3 August to 25 August 2022, to Canada from 25 August to 2 September 2022, and to the Philippines from 2 September to 5 September 2022. He is a man who has sex with men (MSM). He had been sexually active with multiple partners without the use of condom, and practised oral-genital sex. One week prior to admission, he noticed two painless penile ulcers, with subsequent appearance of rash on the face, neck, trunk, and limbs, developing from papules, vesicles then to pustules (Supplementary Figure 1). He enjoyed good past health. On admission, the patient was afebrile, with physical examination revealing bilateral inguinal lymphadenopathy and two painless ulcers at the inner prepuce of the penis. Laboratory testing on admission showed leucocytosis (white blood cells 10.99 × 10^9^/L) and lymphocytosis (lymphocytes 4.51 × 10^9^/L), with atypical lymphocytes up to 26.8%, and elevated alanine transaminase (ALT 61 IU/mL), together with normal renal function test. Abdominal ultrasound revealed no hepatosplenomegaly. Serological tests were negative for blood-borne viruses including hepatitis B surface antigen, hepatitis C virus antibody, and HIV antigen/antibody. Further investigations for infectious mononucleosis including Epstein-Barr virus (EBV) viral capsid antigen IgM, Cytomegalovirus (CMV) IgM, and Toxoplasma IgM/IgG were all negative. Electron microscopy of the vesicular fluid showed brick-shaped virions (Supplementary Figure 2).

Multiple specimens including deep throat saliva, throat swabs, vesicle swabs, rectal swab, urine, and blood were collected on admission, with DNA extraction using EZ1 Virus Mini Kit version 2.0 (QIAGEN, Germany). These were subjected to MPXV real-time polymerase chain reaction (PCR) using in-house assays targeting the TNF receptor gene of MPXV. A plasmid standard was prepared using pCRII-TOPO vector (Invitrogen, USA) cloned with a target insert. A plasmid stock (2 × 10^10^ copies/μL) was diluted in AE buffer to prepare working stocks, which were aliquoted and kept at −80°C. A working stock was further diluted in AE buffer to final concentrations of 2 × 10^5^, 2 × 10^4^, 2 × 10^3^, 2 × 10^2^ and 2 × 10^1^ copies/μL as a quantification standard for the in-house quantitative PCR [6].

The diagnosis of monkeypox was confirmed by the detection of MPXV DNA by PCR in all specimens, with deep throat saliva, throat swabs, and vesicle swabs showing higher viral load when compared with other clinical specimens (Supplementary Table 1).

**Figure 1.**
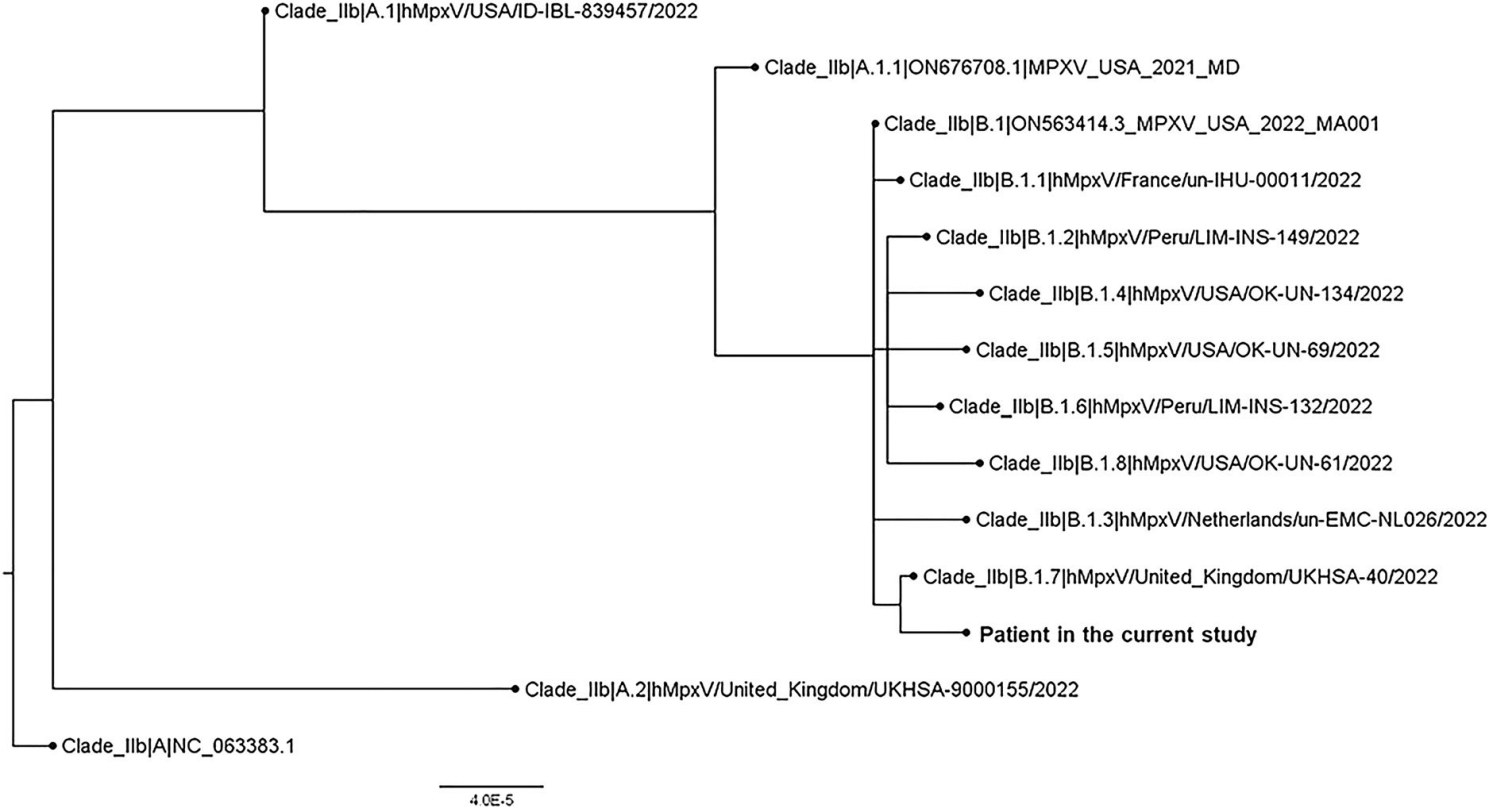
Whole genome phylogenetic analysis of the patients’ strain. The tree was constructed by maximum likelihood method with IQTree2.

Further, whole genome sequencing performed by nanopore sequencing showed that our strain (hMpxV/Hong Kong/HKU-220914-001/2022, GISAID accession no.: EPI_ISL_14945299) belongs to Clade IIb; Lineage B.1.7 (Figure 1). It is most closely related to hMpxV/United_Kingdom/UKHSA-40/2022. Several unique nucleotide mutations were detected in our strain (compared to B.1.7 complete sequences deposited to GISAID as of 13 September 2022; numbering according to NCBI Reference Sequence: NC_063383.1), including C142797T (OPG164:S7L), C149137T (OPG174:D87N), G150706A, G186791A, C188491T, and deletion at nucleotide position 136513–136515 (OPG153:D372-).

As the patient was immunocompetent without any end-organ involvement, antiviral agent was not prescribed to the patient. Serial monitoring of blood tests revealed normalization of atypical lymphocytosis and improvement of liver parenchymal enzymes. Symptoms of sore throat and dysphagia resolved with symptomatic medications. He was discharged from the hospital 2 weeks after admission, with all scabs fallen off and formation of a fresh layer of skin.

## Discussion

Classical monkeypox is characterized by prodromal symptoms of fever, headache, and myalgia together with regional lymphadenopathy and monomorphic rash. The rash develops through different stages ranging from macules, papules, vesicles, and pustules with central umbilication to scabs within 14–21 days, distributing in a centrifugal pattern. Previous studies already reported that the clinical presentations of monkeypox in the current outbreak are atypical, with initial rash in the penile, perianal, and pharyngeal areas depending on the route of exposure, together with less extensive distribution of rash and mild systemic symptoms [7]. With the change in route of exposure in the current outbreak, inflammation of the upper airway including tonsillitis, pharyngitis, epiglottitis, peritonsillar abscess, and retropharyngeal abscess has been described [8,9]. Other complications of human monkeypox include bronchopneumonia, myocarditis, encephalitis, and keratitis with permanent visual loss [8,10].

Leucocytosis and elevated alanine transaminase have been reported in monkeypox [11]. However, our case appears to be the first one presenting as an infectious mononucleosis-like syndrome in the literature. Common causes of infectious mononucleosis include primary infection of EBV, CMV, HIV, and *Toxoplasma gondii*, but these were excluded by serological tests in our case [12]. Currently, it is uncertain whether human monkeypox can present as an infectious mononucleosis-like syndrome without rash, similar to zoster sine herpete. Therefore, monkeypox should be considered as one of the differential diagnoses in patients with infectious mononucleosis, especially for those with a history of epidemiological exposure.

MPXV DNA could be detected in various specimens from patients as reported in the literature, including vesicle swabs, throat swabs, rectal swabs, blood, and urine [8,13]. Our study additionally demonstrated that the viral load in deep throat saliva was comparable to that in vesicle swabs collected from multiple sites. Therefore, deep throat saliva appears to be an alternative clinical specimen for early diagnosis of monkeypox.

## Informed consent

Informed consent has been obtained from the patient.

## Supplementary Material

Supplemental Material
