# Supplementary material for: The first case of monkeypox in Hong Kong presenting as infectious mononucleosis-like syndrome
Source: Emerg Microbes Infect. 2022 Dec 12;12(1):2146910. doi: 10.1080/22221751.2022.2146910 (PMC9718374; doi:10.1080/22221751.2022.2146910)
Supplement: Supplemental Material [file TEMI_A_2146910_SM2377.zip › EMI Supplementary Material.docx]

**Supplementary Materials**

Supplementary Table 1. Cycle threshold value and viral load of monkeypox virus real time PCR in clinical specimens obtained on admission

| Specimen Type | Cycle threshold value using In-house MPXV PCR | Viral load of MPXV (copies/mL) |
| --- | --- | --- |
| Deep throat saliva | 17.95 | 1.02 x 10^8^ |
| Throat swab | 18.79 | 5.85 x 10^7^ |
| Vesicle swab (right face) | 16.55 | 2.60 x 10^8^ |
| Vesicle swab (left arm) | 18.13 | 9.08 x 10^7^ |
| Vesicle swab (upper back) | 19.66 | 3.28 x 10^7^ |
| Rectal swab | 34.06 | 2.24 x 10^3^ |
| Plasma | 29.68 | 4.14 x 10^4^ |
| Serum | 31.45 | 1.27 x 10^4^ |
| Urine | 25.53 | 6.57 x 10^5^ |

Supplementary Table 2. GISAID acknowledgement table

**Supplementary Figure legends**

Supplementary Figure 1: Clinical photo of the forehead of the patient with multiple pustules with erythematous base together with central umbilication and one lesion with scab formation

Supplementary Figure 2: Electron microscopy of MPXV from the vesicular fluid showing brick-shaped virions
