# Supplementary material for: The first case of monkeypox in Hong Kong presenting as infectious mononucleosis-like syndrome
Source: Emerg Microbes Infect. 2022 Dec 12;12(1):2146910. doi: 10.1080/22221751.2022.2146910 (PMC9718374; doi:10.1080/22221751.2022.2146910)
Supplement: Supplemental Material [file TEMI_A_2146910_SM2377.zip › EMI Supplementary Methods.docx]

**SUPPLEMENTARY METHODS**

**Viral nucleic acid extraction**

Clinical specimens (200 μL) were subjected to total nucleic acid (TNA) extraction by EZ1 Virus Mini Kit version 2.0 (QIAGEN, Germany), with the elution volume of 60 μL [1].

**Real-time PCR assays for monkeypox virus detection**

In-house real-time PCR assay for monkeypox virus (MPXV) detection was performed using QuantiNova Probe PCR Kit (QIAGEN). Primers and probe used for the in-house assay were shown in Table 1. Each 20 μL reaction mixture contained 10 μL of 2X QuantiNova Probe PCR Master Mix (QIAGEN), 3 μL of nuclease-free water, 0.8 μL of each 10 μM forward and reverse primer, 0.4 μL of 10 μM probe and 5 μL of TNA. The in-house assay was performed using LightCycler^®^ 96 Real-Time PCR System (Roche). The thermocycling conditions consisted of 95°C for 2 min, followed by 45 cycles of 95°C for 5 s and 55°C for 30 s.

**Standards for viral load measurement**

A plasmid standard was prepared using pCRII-TOPO vector (Invitrogen, USA) cloned with a target insert. A plasmid stock (2×10^10^ copies/μL) was diluted in AE buffer to prepare working stocks, which were aliquoted and kept at -80°C. A working stock was further diluted in AE buffer to final concentrations of 2×10^5^, 2×10^4^, 2×10^3^, 2×10^2^ and 2×10^1^ copies/μL as a quantification standard for the in-house quantitative PCR [2].

**Table 1. Primers and probe used in the present study.**

| **Primer/probe** | | **Sequence (5’ to 3’)** | **Target** | **Reference** |
| --- | --- | --- | --- | --- |
| Forward | GGAAAGTGTAAAGACAACGAATACAG | | MPXV (93bp TNF receptor gene) | Modified from [2] |
| Reverse | CTTGCTATCACATAATCTGAAAGCGT | |  |  |
| Probe | HEX- AAGCCGTAATCTATGTTGTCTATCGTGTCC -lABkFQ | |  |  |
|  |  | |  |  |

bp, base pairs

**Whole genome sequencing for monkeypox virus**

Whole genome sequencing of monkeypox virus was performed using the Oxford Nanopore MinION device (Oxford Nanopore Technologies). The whole genome library was prepared by PCR Tiling methodology using the primer scheme designed by Martin Schou Pedersen (Department of Clinical Microbiology, Rigshospitalet, Copenhagen University Hospital, Copenhagen, Denmark) using Primal Scheme to generate overlapping 2500bp amplicons [3]. Briefly, the extracted DNA was subjected to PCR amplification using the MonkeyPox primer scheme with Q5® Hot Start High-Fidelity 2X Master Mix kit (New England Biolabs, Ipswich, Massachusetts, United States). The PCR conditions were 98°C for 30s for initial denaturation, followed by 35 cycles of 98°C for 15s and 63°C for 5min. The products from the two PCR pools were then combined and purified using 1.5x AMPure XP beads (Beckman Coulter, Brea, CA, USA). The purified PCR product was then quantified using Qubit dsDNA HS Assay Kit (Thermo Fisher Scientific, Waltham, Massachusetts, United States) and the product was examined using gel electrophoresis. Nanopore sequencing library was prepared using the purified PCR product with the Ligation Sequencing Kit XL (SQL-LSK109-XL) following the manufacture instructions and sequenced on the Oxford Nanopore MinION device using R9.4.1 flow cells for 11 hours. For bioinformatics analysis, Medaka was used for generating the consensus sequence. The consensus sequence was aligned with other monkeypox sequences using MAFFT. The phylogenetic tree was constructed using IQTree2, and was visualized using FigTree.

**References for Supplementary Methods**

1. Sridhar S, Yip CCY, Lo KHY, et al. Hepatitis E Virus Species C Infection in Humans, Hong Kong. Clin Infect Dis. 2022 Aug 25;75(2):288-296.

2. Li Y, Zhao H, Wilkins K, et al. Real-time PCR assays for the specific detection of monkeypox virus West African and Congo Basin strain DNA. J Virol Methods. 2010 Oct;169(1):223-7.

3. Welkers M, Jonges M, van den Ouden A. Monkeypox virus whole genome sequencing using combination of NextGenPCR and Oxford Nanopore. [cited 2022 Sep 13]. <https://www.protocols.io/view/monkeypox-virus-whole-genome-sequencing-using-comb-n2bvj6155lk5/v1?step=1>.
