# Supplementary figures and images for: The first case of monkeypox in Hong Kong presenting as infectious mononucleosis-like syndrome
Source: Emerg Microbes Infect. 2022 Dec 12;12(1):2146910. doi: 10.1080/22221751.2022.2146910 (PMC9718374; doi:10.1080/22221751.2022.2146910)

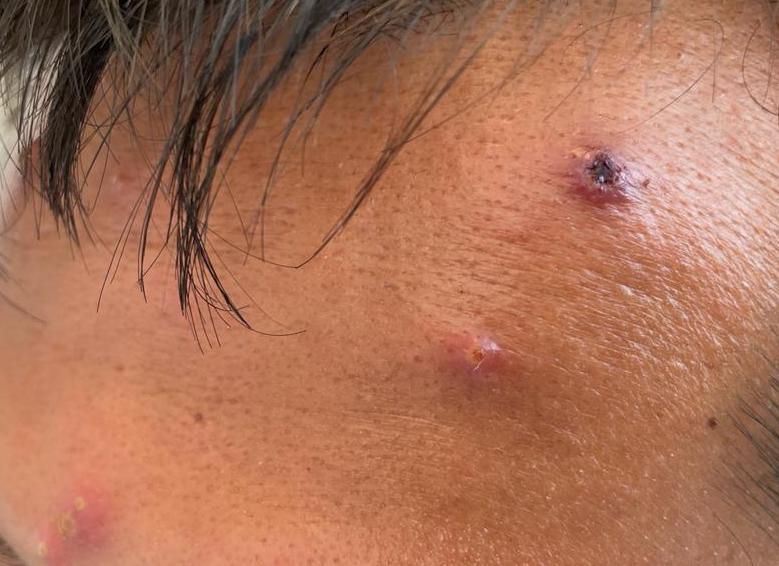

Supplement: Supplemental Material [file TEMI_A_2146910_SM2377.zip › Supplementary Figure 1.jpeg]

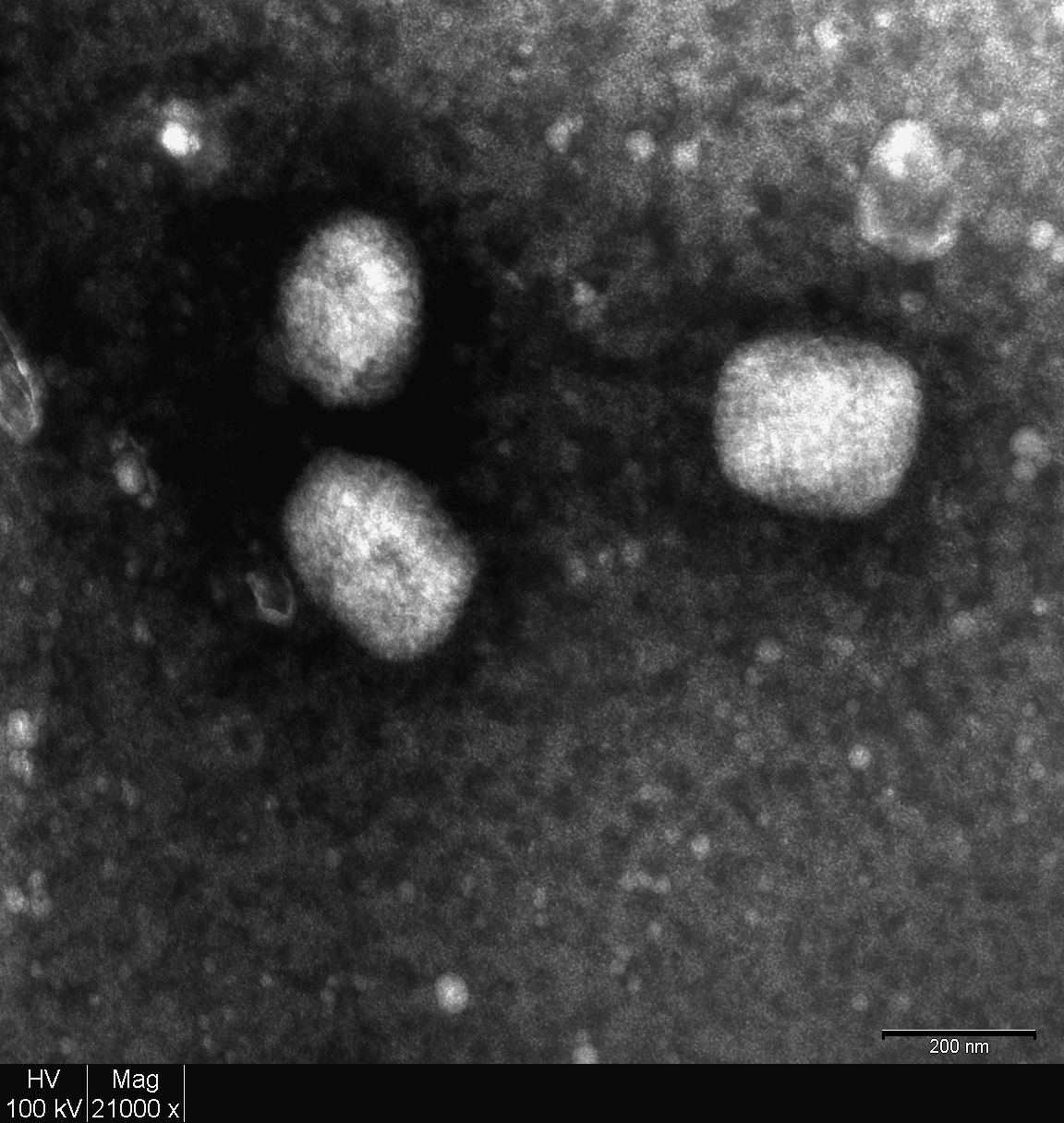

Supplement: Supplemental Material [file TEMI_A_2146910_SM2377.zip › Supplementary Figure 2.TIF]
